# Survival and prognostic factors of non-small cell lung cancer patients with postoperative locoregional recurrence treated with radical radiotherapy

**DOI:** 10.1186/s40880-017-0261-0

**Published:** 2017-12-11

**Authors:** Li Ma, Bo Qiu, Jun Zhang, Qi-Wen Li, Bin Wang, Xu-Hui Zhang, Meng-Yun Qiang, Zhao-Lin Chen, Su-Ping Guo, Hui Liu

**Affiliations:** Department of Radiation Oncology, Sun Yat-sen University Cancer Center, State Key Laboratory of Oncology in South China, Collaborative Innovation Center for Cancer Medicine, 651 Dongfeng Road East, Guangzhou, Guangdong 510060 P. R. China

**Keywords:** Non-small cell lung cancer, Locoregional recurrence, Radical radiotherapy, Biological effective dose, Epidermal growth factor receptor

## Abstract

**Background:**

Locoregional recurrence remains the challenge for long-term survival of non-small cell lung cancer (NSCLC) patients after radical surgery, and curative-intent radiotherapy could be a treatment choice. This study aimed to assess the survival and prognostic factors of patients with postoperative locoregionally recurrent NSCLC treated with radical radiotherapy.

**Methods:**

We reviewed medical records of 74 NSCLC patients with postoperative locoregional recurrence who received radical radiotherapy between April 2012 and February 2016 at Sun Yat-sen University Cancer Center (Guangzhou, China). The efficacy and safety of radical radiotherapy were analyzed. The probability of survival was estimated using the Kaplan–Meier method and compared using the log-rank test. The Cox proportional hazards model was used to identify prognostic factors.

**Results:**

Grade 3/4 adverse events included neutropenia (8 cases, 10.8%), esophagitis (7 cases, 9.5%), pneumonitis (1 case, 1.4%), and vomiting (1 case, 1.4%). The 2-year overall survival, progression-free survival, local recurrence-free survival (LRFS), and distant metastasis-free survival (DMFS) rates of all patients were 84.2, 42.5, 70.0, and 50.9%, respectively. Univariate and multivariate analyses showed that a higher biological effective dose (BED) of radiation was associated with longer LRFS [hazard ratios (HR) = 0.317, 95% confidence interval (CI) = 0.112–0.899, *P* = 0.016] and that wild-type epidermal growth factor receptor (*EGFR*) was associated with longer DMFS compared with *EGFR* mutation (HR = 0.383, 95% CI = 0.171–0.855, *P* = 0.019).

**Conclusions:**

Radical radiotherapy is effective and well-tolerated in NSCLC patients with postoperative locoregional recurrence. High BED is a predictor for long LRFS, and the presence of wild-type *EGFR* is a predictor for long DMFS.

## Introduction

Lung cancer remains the most common cancer and the first leading cause of cancer-related death worldwide [1]. For patients with early-stage (stages I and II) and resectable stage III. A non-small cell lung cancer (NSCLC), the National Comprehensive Cancer Network (NCCN) guidelines recommend surgical resection to be the primary treatment as it offers a chance of cure. The 5-year overall survival (OS) rates of patients who had undergone complete tumor resection range from 24 to 73% [2, 3], and postoperative recurrence remains the challenge for long-term survival. The recurrence rates of patients who have undergone surgery range from 34 to 45% [4–7]. Patterns of failure include distant metastasis, locoregional recurrence, and distant metastasis with locoregional recurrence [8]. Locoregional recurrence occurs in 20–45% patients [5, 9–11].

Unlike the patients with distant failure, the patients with locoregional recurrence after radical surgery without hematogenous spreading can be cured by sufficient local treatment [12]. Most patients with locoregionally recurrent disease cannot bear secondary radical surgery, which highlights the importance of radiotherapy for curative purpose. In the previous studies, radiotherapy has been reported to achieve a promising outcome in patients with locoregionally recurrent NSCLC, with a median OS of 17 to 37.3 months [12–16]. However, most of these studies had small sample sizes. In clinical practice, the standard treatment has not been established regarding the radiation dose or whether in combination with chemotherapy. Moreover, the prognostic factors and failure patterns after radical radiotherapy are also worthy of exploration. In the present study, we evaluated the survival, prognostic factors, and failure patterns after radical radiotherapy for patients with postoperative locoregional recurrence of NSCLC.

## Patients and methods

### Patient selection and acquisition of clinical data

NSCLC patients with postoperative locoregional recurrence treated with radical radiotherapy with or without concurrent chemotherapy between April 2012 and February 2016 at Sun Yat-sen University Cancer Center (Guangzhou, China) were screened. Patient selection criteria were as follows: (1) R0 resection (no residual microscopic disease) for primary NSCLC; (2) postoperative recurrence proved by biopsy or confirmed by imaging modalities and subsequent clinical outcomes; (3) recurrent disease within the ipsilateral hemithorax, mediastinum, and/or supraclavicular fossa; and (4) no metastases to solid organs, the pleura, or the peritoneum. Patients who had previous or recent another malignancy or Eastern Cooperative Oncology Group (ECOG) performance status > 2 were excluded.

Clinical data collected from each patient included age, sex, thoracic surgery history, stage and histology of primary NSCLC, epidermal growth factor receptor (*EGFR*) mutation status, interval between surgery and recurrence, and recurrence sites. The 7th edition of the American Joint Committee on Cancer (AJCC) staging system for lung cancer was used to stage the primary tumor.

### Ethical statement

The treatment of all patients was discussed by the thoracic multi-disciplinary treatment team, and participant information collection was approved by the Ethics Committee of Sun Yat-sen University Cancer Center. Written informed consent was obtained from patients for the use of their data in clinical research.

### Radical radiotherapy

Patient immobilization, simulation, and treatment planning were performed according to the standard protocol of radiotherapy for lung cancer in Sun Yat-sen University Cancer Center [17]. With the patient in the supine position, a cradle for immobilization was made with vacuum. Using 4-dimensional computed tomography (4D-CT), 3-dimensional (3D) data sets associated with 10 respiratory phases in 0.5-cm thickness slices were constructed, and a maximum intensity projection data set was generated. All 11 data sets were exported to the Monaco planning system (Elekta Medical Systems, Stockholm, Sweden) for target contouring and treatment planning. Individual patient was scanned from the atlas (C1) level to the second lumbar vertebra (L2) level to cover the whole neck and lungs. Briefly, the gross tumor volume (GTV-lung plus GTV-lymph node) consisted of recurrent lesion diagnosed by biopsy or subsequent CT scan; the regions of tumor revealed by endoscopy but not seen on CT images were also included in the GTV-lung. The clinical target volume (CTV) comprised a 0.6-cm margin around GTV-lung and selected lymph node region. CTV was not necessary for stereotactic body radiotherapy (SBRT). Two planning target volume (PTV) had been defined: PTV1 was defined as the GTV plus a 0.6-cm margin, and PTV2 was defined as the CTV plus a 0.6-cm margin in all directions. The intensity-modulated radiotherapy (IMRT) or 3-dimensional conventional radiotherapy (3D-CRT) technique was used to deliver a median dose of 60 Gy (range 56–68 Gy) to PTV1, and 46 Gy (range 40–54 Gy) to PTV2 in a median of 26 fractions (range 15–35 fractions). SBRT was performed to deliver a median dose of 50 Gy (range 50–70 Gy) in a median of 10 fractions (range 10–12 fractions).

### Concurrent chemotherapy

The regimens of concurrent chemotherapy mostly included platinum-based dual drugs: (1) weekly paclitaxel/docetaxel (25 mg/m^2^) and cisplatin/nedaplatin (25 mg/m^2^); and (2) pemetrexed (500 mg/m^2^) and cisplatin/nedaplatin (75 mg/m^2^) every 3 weeks. Single-agent treatment included pemetrexed (500 mg/m^2^), taxel (175 mg/m^2^), and 5-fluorouracil (2.75 g/m^2^) every 3 weeks.

### Follow-up

The follow-up started since the first day of radiotherapy and ended on May 31, 2017. Tumor response was assessed 2 months after radical radiotherapy according to the Response Evaluation Criteria In Solid Tumors (RECIST). The responses included complete response (CR), partial response (PR), progressive disease (PD), and stable disease (SD). The adverse events were graded according to the Common Toxicity Criteria for Adverse Events version 4.0 (CTCAE 4.0) from the start of radiotherapy until 1 year afterward. Patients underwent chest and upper abdominal CT scan every 3 months and brain magnetic resonance imaging (MRI) every 6 months for the first 2 years; chest and upper abdominal CT and brain MRI every 6 months for another 3 years, and then annually. Whole-body bone scan was performed when patients were suspected for bone metastasis.

Locoregional recurrence was defined as disease recurrence at the surgical margin, ipsilateral hemithorax, or regional lymph nodes. Metastasis to the contralateral lung and to outside of the hemithorax or mediastinum was defined as distant metastasis. OS was defined as the duration from the first day of radical radiotherapy to the date of death from any cause or to the last visit before May 31, 2017. Patients were censored at the date of last follow-up if alive or lost to follow-up. Progression-free survival (PFS), locoregional recurrence-free survival (LRFS), and distant metastasis-free survival (DMFS) were defined as the interval from the first day of radical radiotherapy to documented disease progression, locoregional recurrence, and distant metastasis, respectively. Patients were censored at the date of death or the date of last follow-up with no evidence of indicated events.

### Statistical analysis

The probability of survival was estimated using the Kaplan–Meier method and compared using the log-rank test. The multivariate Cox proportional hazards model was used to estimate hazard ratios (HRs) and 95% confidence intervals (CIs) for the chance of survival. Age, sex, stage of primary tumor, histology, recurrence site, interval between surgery and recurrence, biological effective dose (BED) of radiation, concurrent chemotherapy, *EGFR* status, irradiation technique, and GTV were included in univariate analysis. All tests were two-sided. Univariate variables that reached a *P* value < 0.2 were evaluated in multivariate analysis. *P* values less than 0.05 were considered significant. All statistical analyses were performed using SPSS 22.0 software (IBM, Chicago, IL, USA).

## Results

### Patient characteristics

We identified 74 patients comprising 20 females and 54 males. The median age of patients was 59 years (range 29–84 years). Patient characteristics are summarized in Table 1. The information of pathologic stage of tumor confirmed after initial surgery was available for 67 patients, 34 of whom had stage III disease. Information of *EGFR* status was available for 51 patients, of which 14 had *EGFR* mutation. The median interval between surgery and recurrence was 11 months (range 6–104 months). Regional lymph nodes were the most common site for recurrence [56 cases (75.7%)].

**Table 1:** Characteristics of 74 NSCLC patients with postoperative locoregional recurrence treated with radical radiotherapy

Characteristic	Number of patients (%)
Sex	
Male	54 (73.0)
Female	20 (27.0)
Stage of primary tumor^a^	
I	17 (23.0)
II	16 (21.6)
III	34 (45.9)
Unknown	7 (9.5)
Histology	
Squamous cell carcinoma	18 (24.3)
Adenocarcinoma	41 (55.4)
Lymphoepithelioma-like carcinoma	10 (13.3)
Others	5 (6.8)
*EGFR* status	
Mutation	14 (18.9)
Wild-type	37 (50.0)
Unknown	23 (31.1)
Recurrence site	
Stump	8 (10.8)
Regional lymph nodes	56 (75.7)
Ipsilateral lung	6 (8.1)
Both stump and regional lymph nodes	3 (4.0)
Both regional lymph nodes and ipsilateral lung	1 (1.4)
Concurrent chemotherapy	
Yes	59 (79.7)
No	15 (20.3)
Radiation technique	
3D-CRT	5 (6.8)
IMRT	62 (83.8)
SBRT	7 (9.4)

*NSCLC* non-small cell lung cancer, *EGFR* epidermal growth factor receptor, *3D-CRT* 3-dimensional conformal radiotherapy, *IMRT* intensity-modulated radiotherapy, *SBRT* stereotactic body radiotherapy

^a^The 7th edition of the American Joint Committee on Cancer (AJCC) staging system was used

Most patients underwent IMRT. The median GTV was 28.7 cm^3^ (range 2.6–189.3 cm^3^). The median BED of radiation was 78 Gy (range 55–119 Gy). Fifty-nine (79.7%) patients underwent concurrent chemotherapy: 52 (70.3%) were treated with platinum-based dual drugs, and 7 (9.4%) underwent single-agent treatment. Eighteen (24.3%) patients underwent neoadjuvant chemotherapy for recurrent disease before radiotherapy.

### Adverse events

The average weight loss was 0.8 kg (range 0–6.5 kg). The most frequent adverse events observed were neutropenia, esophagitis, pneumonitis, and vomiting, and most were mild (grades 1 and 2). Grade 3 neutropenia occurred in 8 (10.8%) patients, followed by grade 3/4 esophagitis in 7 (9.5%) patients, grade 3 pneumonitis in 1 (1.4%) patient, and grade 3 vomiting in 1 (1.4%) patient. Grade 4 esophagitis occurred in 1 (1.4%) patient, who developed malignant fistulae. There were no treatment-related deaths.

### Treatment outcome

**Fig. 1:**
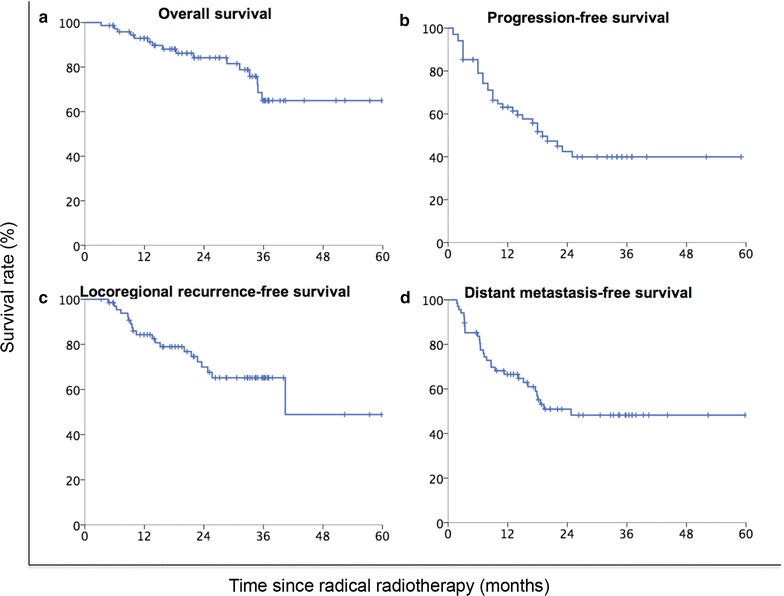
Kaplan–Meier survival curves of non-small cell lung cancer (NSCLC) patients with postoperative locoregional recurrence treated with radical radiotherapy. **a** Overall survival curve; **b** progression-free survival curve; **c** locoregional recurrence-free survival curve; **d** distant metastasis-free survival curve

Two months after radical radiotherapy, 11 (14.9%) patients achieved CR, 36 (48.6%) achieved PR, 14 (18.9%) had SD, and 13 (17.6%) had PD. With a median follow-up of 21.5 months (range 2–50 months), the 2-year OS, PFS, LRFS, and DMFS rates of all patients were 84.2, 42.5, 70.0, and 50.9%, respectively. Sixteen patients died during follow-up. The median estimated OS was not reached. The median estimated PFS, LRFS, and DMFS were 19.0, 40.4, and 24.8 months, respectively (Fig. 1).

Univariate and multivariate analyses showed that higher BED of radiation was associated with longer LRFS (HR = 0.317, 95% CI = 0.112–0.899, *P* = 0.016) and that the presence of wild-type *EGFR* was associated with longer DMFS compared with *EGFR* mutations (HR = 0.383, 95% CI = 0.171–0.855, *P* = 0.019) (Tables 2, 3).

**Table 2:** Univariate analyses of prognostic factors for 74 NSCLC patients with postoperative locoregional recurrence treated with radical radiotherapy

Variable	OS		PFS		LRFS		DMFS	
	HR (95% CI)	*P* value	HR (95% CI)	*P* value	HR (95% CI)	*P* value	HR (95% CI)	*P* value
Age (≥ 59 vs. < 59 years)	1.387 (0.517–3.723)	0.516	0.532 (0.264–1.071)	0.077	0.789 (0.322–1.932)	0.604	0.619 (0.296–1.292)	0.201
Sex (male vs. female)	0.694 (0.197–2.443)	0.570	1.234 (0.603–2.527)	0.565	0.898 (0.324–2.492)	0.837	1.286 (0.605–2.735)	0.513
Stage of primary tumor (I vs. II vs. III)	1.308 (0.700–2.447)	0.400	1.173 (0.775–1.775)	0.451	1.084 (0.643–1.827)	0.762	1.189 (0.758–1.863)	0.451
Histology (squamous cell carcinoma vs. non-squamous cell carcinoma)	0.755 (0.261–2.183)	0.604	1.125 (0.510–2.482)	0.770	0.750 (0.270–2.085)	0.582	1.420 (0.582–3.466)	0.441
Recurrence site (stump vs. regional lymph nodes vs. ipsilateral lung vs. multiple sites)	1.292 (0.689–2.423)	0.425	1.284 (0.767–2.149)	0.342	1.439 (0.780–2.655)	0.245	1.433 (0.840–2.445)	0.187
Interval between surgery and recurrence (≥ 10 vs. < 10 months)	0.882 (0.328–2.373)	0.803	0.690 (0.352–1.351)	0.279	1.054 (0.434–2.559)	0.908	0.655 (0.320–1.339)	0.246
BED of radiation (≥ 78 vs. < 78 Gy)	0.347 (0.119–1.013)	0.053	0.905 (0.466–1.758)	0.769	0.288 (0.104–0.795)	0.016	1.075 (0.531–2.175)	0.841
Concurrent chemotherapy (yes vs. no)	1.222 (0.348–4.294)	0.754	1.114 (0.485–2.555)	0.799	1.247 (0.412–3.774)	0.696	1.150 (0.471–2.808)	0.758
*EGFR* status (wild-type vs. mutation type vs. unknown)	0.265 (0.063–1.112)	0.069	0.509 (0.238–1.088)	0.509	1.694 (0.465–6.175)	0.425	0.418 (0.192–0.912)	0.028
Radiation technique (3D-CRT vs. IMRT vs. SBRT)	2.562 (0.887–7.395)	0.082	1.213 (0.554–2.654)	0.630	2.047 (0.757–5.537)	0.158	1.130 (0.472–2.701)	0.784
GTV (≥ 28.7 vs. < 28.7 cm^3^)	1.864 (0.637–5.455)	0.256	1.246 (0.593–2.617)	0.561	0.543 (0.201–1.470)	0.230	1.525 (0.682–3.411)	0.304

*OS* overall survival, *PFS* progression-free survival, *LRFS* local recurrence-free survival, *DMFS* distant metastasis-free survival, *HR* hazard ratio, *CI* confidence interval, *BED* biological effective dose, *EGFR* epidermal growth factor receptor, *3D-CRT* 3-dimensional conformal radiotherapy, *IMRT* intensity-modulated radiotherapy, *SBRT* stereotactic body radiotherapy, *GTV* gross tumor volume

**Table 3:** Multivariate analyses of prognostic factors for 74 NSCLC patients with postoperative locoregional recurrence treated by radical radiotherapy

Variable	OS		PFS		LRFS		DMFS	
	HR (95% CI)	*P* value	HR (95% CI)	*P* value	HR (95% CI)	*P* value	HR (95% CI)	*P* value
Recurrence site (stump vs. regional lymph nodes vs. ipsilateral lung vs. multiple sites)	–		–		–		1.421 (0.802–2.519)	0.229
BED of radiation (≥ 78 vs. < 78 Gy)	0.472 (0.070–3.182)	0.472	–		0.317 (0.112–0.899)	0.031	–	
EGFR status (wild-type vs. mutation type vs. unknown)	0.227 (0.034–1.498)	0.118	–		–		0.383 (0.171–0.855)	0.019
Radiation technique (3D-CRT vs. IMRT vs. SBRT)	4.406 (0.667–29.085)	0.124	–		1.525 (0.516–4.503)	0.445	–	

*OS* overall survival, *PFS* progression free survival, *LRFS* local recurrence free survival, *DMFS* distant metastasis free survival, *HR* hazard ratio, *CI* confidence interval, *BED* biological effective dose, *EGFR* epidermal growth factor receptor, *3D-CRT* 3-dimensional conformal radiotherapy, *IMRT* intensity-modulated radiotherapy, *SBRT* stereotactic body radiotherapy, *GTV* gross tumor volume, *–* not included

### Failure pattern

During follow-up, 35 (47.3%) patients experienced disease progression. The most common failure pattern was locoregional recurrence plus distant metastasis, observed in 16 (21.6%) patients. Fifteen (20.3%) patients had distant metastasis alone, and 4 (5.4%) had locoregional recurrence alone. The contralateral lung (14 cases, 18.9%) was the most common site of metastasis, followed by the brain (5 cases, 6.8%), liver (4 cases, 5.4%), bone (3 cases, 4.1%), adrenal gland (2 cases, 2.7%), celiac lymph nodes (2 cases, 2.7%), and axillary lymph nodes (1 case, 1.4%). Among the 20 patients with locoregional progression, 7 (35.0%) had progression at the previous recurrence sites.

## Discussion

The present study demonstrated that radical radiotherapy was effective and well-tolerated in NSCLC patients with postoperative locoregional recurrence. The 2-year OS, PFS, LRFS, and DMFS rates of all patients were 84.2, 42.5, 70.0, and 50.9%, respectively. We evaluated the possible prognostic roles of age, sex, interval between surgery and recurrence, BED of radiation, concurrent chemotherapy, *EGFR* status, and GTV. High BED appeared to be an independent predictor for prolonged LRFS. Patients with wild-type *EGFR* were found to have longer DMFS than those with *EGFR* mutation.

No independent predictors for OS were identified in the present study. In univariate analysis, BED showed a trend to be associated with OS (*P* = 0.053). However, the difference was not significant in multivariate analysis (*P* = 0.472). The prognostic factors for OS of patients with local recurrence were also investigated in several retrospective studies. Female [7, 18], young age [7], long disease-free interval between initial surgery and local recurrence [7, 15], and high radiation dose prescribed [16] were reported to be associated with prolonged OS. Early stage of primary tumor [18] and recurrent lesion [15, 18] as well as recurrence in the bronchial stump [16, 18–20] were also reported to be associated with prolonged OS.

Several studies have demonstrated that BED of radiation was associated with local control in NSCLC patients receiving radiotherapy [21–23]. After analyzing potential prognostic factors, we found that higher BED was an independent indicator for longer LRFS (*P* = 0.031) in the present study. Patients receiving a BED of radiation no less than 78 Gy had a longer LRFS than did those receiving a BED less than 78 Gy. Therefore, escalating BED might be helpful to patients with local recurrence. However, the volume of the lungs of NSCLC patients was decreased with reduced pulmonary function after surgery. Some patients had received several cycles of adjuvant or neoadjuvant chemotherapy, which resulted in poor performance status. Therefore, the lung toxicity accompanied with high BED warrants attention, and the dose constraints for normal tissues should be followed strictly. In the present study, a mean lung dose less than 17 Gy and the lung volumes irradiated above 20 Gy (V_20_) less than 30% were constrained. Only 1 patient had grade 3 pneumonitis. No grade 4/5 lung adverse event was found.

As we know, concurrent chemotherapy prolonged survival of patients with unresectable stage III NSCLC [24–29]. For patients with postoperative local recurrence, the role of concurrent chemotherapy has not been fully elucidated [14, 30]. Our results did not support the prognostic role of chemotherapy for survival. However, this result must be interpreted with caution. Among the 14 patients who did not receive concurrent chemotherapy, 5 were treated with SBRT. The high BED of SBRT could achieve good local control in the absence of concurrent chemotherapy. Therefore, the inclusion of patients receiving SBRT might dilute the role of chemotherapy in the entire cohort. Moreover, our data showed that the major failure pattern after radical radiotherapy was distant metastasis, consistent with the results reported by Kelsey et al. [15], which revealed that 50% patients developed distant metastases after salvage radiotherapy. The high rate of distant metastasis after radical radiotherapy indicates that chemotherapy is important for NSCLC patients with locoregional recurrence. Therefore, the value of chemotherapy concurrent with or after radiotherapy is worthy of further exploration.

Data from multiple randomized trials have demonstrated that *EGFR* mutation may be used as a predictive factor for tumor response to tyrosine kinase inhibitors (TKIs) and biomarkers for TKI treatment selection [31–33]. NSCLC patients with postoperative locoregional recurrence who harbored *EGFR* gene mutations were also reported to benefit from TKIs [34]. In the present study, our results showed that *EGFR*-mutated NSCLC patients were more likely to have distant failure after radical radiotherapy as compared with those with wild-type *EGFR* (*P* = 0.019). Our previous study investigated the association of *EGFR* mutation status with treatment outcome of patients with stage III NSCLC who had undergone a complete (R0) resection, and found that patients with mutant *EGFR* had a higher rate of multiple distant metastases after surgery than those with wild-type *EGFR* [35]. However, *EGFR* mutation status was not found to be a prognostic factor for OS in either our previous study or the present study. The reason may be that *EGFR*-mutated patients benefited from subsequent TKI treatment after distant failure. *EGFR* mutation seems to be more like a predictive marker for TKI treatment than a prognostic marker for OS.

The safety of radiotherapy for NSCLC patients with postoperative locoregional recurrence has been demonstrated by other researchers [12–14, 16]. Similarly, the toxicities were acceptable in the present study. Grade 3 adverse events including pneumonitis, neutropenia, vomiting, and esophagitis appeared in 16 patients. Only one developed grade 4 radiation-related esophagitis (esophagus fistula).

Our results demonstrated that NSCLC patients with postoperative locoregional recurrence could benefit from radical radiotherapy. However, this study had several limitations, such as the short follow-up, small sample size, and a retrospective nature. The results should be confirmed in larger prospective studies.

## Conclusions

Radical radiotherapy is effective and well-tolerated in NSCLC patients with postoperative locoregional recurrence. High BED of radiation appeared to be an independent favorable factor for LRFS. Patients with wild-type *EGFR* were found to have longer DMFS than those with *EGFR* mutations. Distant failure accounted for the substantial treatment failure after radical radiotherapy. The values of chemotherapy concurrent with or after radiotherapy should be further explored.
